# Microwave-induced orbital angular momentum transfer

**DOI:** 10.1038/s41598-019-40195-4

**Published:** 2019-03-05

**Authors:** Zahra Amini Sabegh, Mohammad Ali Maleki, Mohammad Mahmoudi

**Affiliations:** Department of Physics, University of Zanjan, University Blvd., 45371-38791 Zanjan, Iran

## Abstract

The microwave-induced orbital angular momentum (OAM) transfer from a Laguerre-Gaussian (LG) beam to a weak plane-wave is studied in a closed-loop four-level ladder-type atomic system. The analytical investigation shows that the generated fourth field is an LG beam with the same OAM of the applied LG field. Moreover, the microwave-induced subluminal generated pulse can be switched to the superluminal one only by changing the relative phase of applied fields. It is shown that the OAM transfer in subluminal regime is accompanied by a slightly absorption, however, it switches to the slightly gain in superluminal regime. The transfer of light’s OAM and control of the group velocity of the generated pulse can prepare a high-dimensional Hilbert space which has a major role in quantum communication and information processing.

## Introduction

In the past three decades, much attention has been paid to the study of the optical phenomena using Laguerre-Gaussian (LG) laser fields which are derived by solving the Helmholtz equation in the cylindrical coordinates. The intensity profile of the LG beam has a doughnut-shaped pattern whose origin is the phase singularity and the azimuthal component of the beam’s Poynting vector. It is well known that a light beam may have both the orbital angular momentum (OAM) due to its helical wavefront and the spin angular momentum associated with the circular polarization^1,2^. Allen *et al*., for the first time, introduced a well-defined OAM for an LG laser light by measuring the induced mechanical torque on the suspended cylindrical lenses due to the OAM transfer^3^. The LG modes can be practically generated in several common methods such as forked diffraction gratings^4^, computer-generated holograms^5^, cylindrical lens mode converters^6^, spiral phase plates^7^, and spatial light modulators (SLMs)^8^. Light beams with OAM were strongly used in fundamental physical subjects like nonlinear optical phenomena, angular momentum conservation^9^, quantum effects^10^, nano optics^11^, imaging^12^, and quantum communications^13^.

In recent years, the LG beams and their features have been extensively investigated in many aspects. The spatially dependent EIT has been demonstrated by probing cold rubidium atoms with optical vortex light^14^. Generation of the optical vortex beams from a non-vortex beam occurred by a wave mixing process in a nonlinear photonic crystal^15^. Several nonlinear optical processes have been studied in different quantum systems using LG beams^16^ such as the second-harmonic generation^9,17^, sum frequency generation^18^, and four-wave mixing (FWM)^19–22^. The spatially structured optical transparency is theoretically investigated in a five-level combined tripod and Λ atom-light coupling scheme^23^. Mahmoudi *et al*. have reported the spatially dependent atom-photon entanglement using LG laser beams. It was shown that the atom-photon entanglement can be controlled by OAM of light in closed-loop atomic systems^24^. The group velocity of the LG beams in the free space has been also studied. Recently, Boyd *et al*. have shown that the group velocity of a twisted light depends on the distance across the light propagation direction in vacuum^25^. In another work, the subluminal group velocity of the LG beam was reported and it was shown that an optical vortex beam is dispersed even in the free space^26^.

On the other hand, the OAM transfer from a pump field to both probe and generated fields at the FWM frequency was suggested in a two-level system^27^. In the following, it was shown that the OAM transfer from two vortex control fields to a probe field occurs in both four-level tripod-type and double tripod-type atomic systems^28,29^. Recently, the transfer of the optical vortex in four-level double-Λ EIT scheme, through a FWM process, has been reported only by one vortex control field^22^.

In this paper, we are going to introduce a model in which the microwave field induces an OAM transfer from strong coupling LG field to the generated fourth field (GFF). We consider a closed-loop four-level ladder-type atomic system and investigate the effect of the different parameters on the OAM transfer, as well as the intensity and phase profiles. In addition, we investigate the spatially dependent dispersion and absorption of the GFF and obtain an analytical expression to explain the group velocity behavior of the GFF. It is shown that a weak microwave probe field transfers the OAM of a strong coupling LG field to the GFF. The OAM transfer has been already reported just with the absorption-assisted EIT window. Here, we find a new type of the OAM exchange which is ensured by the microwave-induced gain-assisted EIT window. Due to the different scattering processes of the applied fields in the closed-loop quantum systems, the OAM can be transferred from an applied LG field to another laser field and can be controlled by the relative phase of the applied fields. The GFF is an LG field and can superluminally propagate passing through the atomic vapor cell, for the special set of parameters. Thus, our model proposes a simple method to transfer the OAM from an LG beam to a weak plane-wave using the microwave field in the gain-assisted EIT window. Moreover, the slope of dispersion of microwave-induced GFF switches from positive to negative only by changing the relative phase of the applied fields. It is worth to note that, because of the degree of freedom for OAM, the OAM transfer and switching of GFF group velocity can be used for transfer, storage, and processing of high-dimensional optical information.

## Theoretical framework

**Figure 1:**
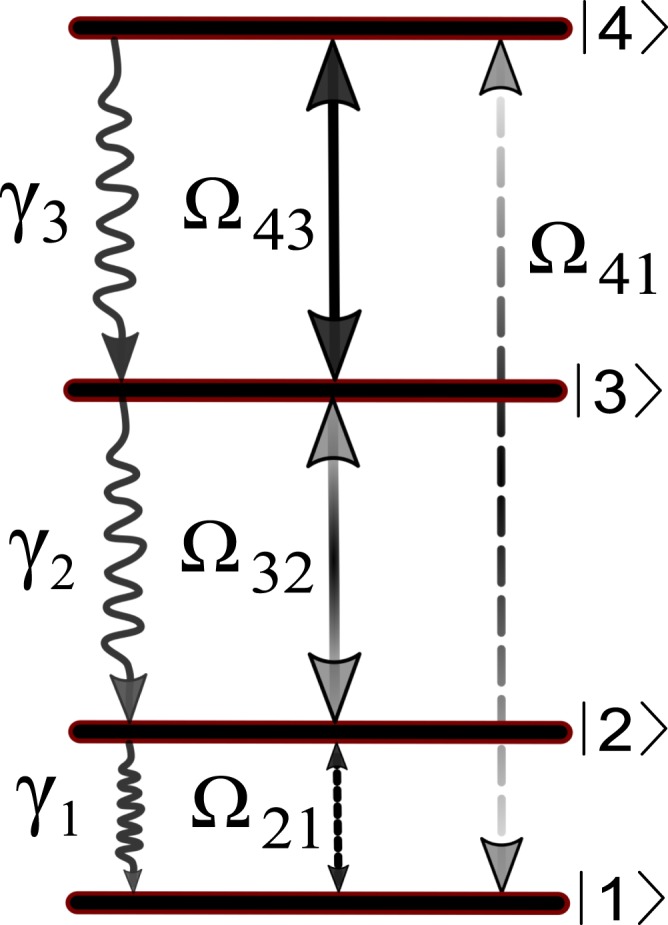
Schematics of the closed-loop four-level ladder-type atomic system which can be established in ^87^*Rb* applying a weak planar microwave field, Ω_21_, strong coupling LG field, Ω_32_, strong coupling planar field, Ω_43_, and weak planar field, Ω_41_. The spontaneous emission rates are indicated by *γ*_1_, *γ*_2_ and *γ*_3_.

The proposed four-level ladder-type atomic system consisting of four energy states is illustrated in Fig. 1. We consider an ensemble of ^87^*Rb* atoms as a realistic example with |1〉 = |5^2^*S*_1/2_, *F* = 1〉, |2〉 = |5^2^*S*_1/2_, *F* = 2〉, |3〉 = |5^2^*P*_3/2_〉, and |4〉 = |7^2^*S*_1/2_〉. The transition |*i*〉 ↔ |*j*〉 is driven by an external field with frequency *ω*_*ij*_ and Rabi frequency ${{\rm{\Omega }}}_{ij}={\overrightarrow{\mu }}_{ij}.{\overrightarrow{E}}_{ij}/\hslash$ (*i*, *j* ∈ 1, ..., 4). Here, *μ*_*ij*_ and *E*_*ij*_ are the induced dipole moment of the transition |*i*〉 ↔ |*j*〉 and the amplitude of the applied field, respectively. A planar microwave field is applied to the |1〉 ↔ |2〉 transition as the first weak probe field. The transition |2〉 ↔ |3〉 is excited by a strong coupling LG field which in the cylindrical coordinates has the form

1 $${E}_{32}(r,\phi )={E}_{{0}_{32}}\frac{1}{\sqrt{|l|!}}{(\frac{\sqrt{2}r}{{w}_{LG}})}^{|l|}\,{e}^{-{r}^{2}/{w}_{LG}^{2}}\,{e}^{i[\frac{n{\omega }_{32}}{c}(z+\frac{{r}^{2}z}{2({z}^{2}+{z}_{R}^{2})})-(|l|+1)ta{n}^{-1}(z/{z}_{R})+l\phi ]},$$

where the field strength, LG beam waist, OAM, refractive index of medium, light frequency, velocity of light in vacuum, and propagation direction of light are denoted by ${E}_{{0}_{32}}$, *w*_*LG*_, *l*, *n*, *ω*_32_, *c*, and *z*, respectively, and ${z}_{R}=n{\omega }_{32}{w}_{LG}^{2}\mathrm{/2}c$ being the Rayleigh range. Two planar fields are exerted to the |3〉 ↔ |4〉 and |1〉 ↔ |4〉 transitions as the second strong coupling and weak probe fields.

The spontaneous emission rates for the transitions |1〉 ↔ |2〉, |2〉 ↔ |3〉 and |3〉 ↔ |4〉 are indicated by *γ*_1_, *γ*_2_ and *γ*_3_. In the rotating-wave and electric-dipole approximations, the time evolution of this system can be described by the density matrix equations of motion which stand for

2 $$\begin{array}{rcl}{\dot{\rho }}_{11} & = & {\gamma }_{1}{\rho }_{22}+i{{\rm{\Omega }}}_{21}^{\ast }{\rho }_{21}-i{{\rm{\Omega }}}_{21}{\rho }_{12}+i{{\rm{\Omega }}}_{41}^{\ast }{\rho }_{41}-i{{\rm{\Omega }}}_{41}{\rho }_{14},\\ {\dot{\rho }}_{22} & = & -{\gamma }_{1}{\rho }_{22}+{\gamma }_{2}{\rho }_{33}+i{{\rm{\Omega }}}_{21}{\rho }_{12}\\  &  & -i{{\rm{\Omega }}}_{21}^{\ast }{\rho }_{21}+i{{\rm{\Omega }}}_{32}^{\ast }{\rho }_{32}-i{{\rm{\Omega }}}_{32}{\rho }_{23},\\ {\dot{\rho }}_{33} & = & -{\gamma }_{2}{\rho }_{33}+{\gamma }_{3}{\rho }_{44}+i{{\rm{\Omega }}}_{32}{\rho }_{23}\\  &  & -i{{\rm{\Omega }}}_{32}^{\ast }{\rho }_{32}+i{{\rm{\Omega }}}_{43}^{\ast }{e}^{-i{\varphi }_{0}}{\rho }_{43}-i{{\rm{\Omega }}}_{43}{e}^{i{\varphi }_{0}}{\rho }_{34},\\ {\dot{\rho }}_{12} & = & -(i{{\rm{\Delta }}}_{21}+{\gamma }_{1}\mathrm{/2)}{\rho }_{12}-i{{\rm{\Omega }}}_{32}{\rho }_{13}\\  &  & +i{{\rm{\Omega }}}_{21}^{\ast }({\rho }_{22}-{\rho }_{11})+i{{\rm{\Omega }}}_{41}^{\ast }{\rho }_{42},\\ {\dot{\rho }}_{13} & = & -[i({{\rm{\Delta }}}_{21}+{{\rm{\Delta }}}_{32})+{\gamma }_{2}\mathrm{/2]}{\rho }_{13}+i{{\rm{\Omega }}}_{21}^{\ast }{\rho }_{23}\\  &  & -i{{\rm{\Omega }}}_{32}^{\ast }{\rho }_{12}-i{{\rm{\Omega }}}_{43}{e}^{i{\varphi }_{0}}{\rho }_{14}+i{{\rm{\Omega }}}_{41}^{\ast }{\rho }_{43},\\ {\dot{\rho }}_{14} & = & -[i{{\rm{\Delta }}}_{41}+{\gamma }_{3}\mathrm{/2]}{\rho }_{14}-i{{\rm{\Omega }}}_{43}^{\ast }{e}^{-i{\varphi }_{0}}{\rho }_{13}\\  &  & +i{{\rm{\Omega }}}_{21}^{\ast }{\rho }_{24}+i{{\rm{\Omega }}}_{41}^{\ast }({\rho }_{44}-{\rho }_{11}),\\ {\dot{\rho }}_{23} & = & -[i{{\rm{\Delta }}}_{32}+({\gamma }_{1}+{\gamma }_{2}\mathrm{)/2]}{\rho }_{23}+i{{\rm{\Omega }}}_{21}{\rho }_{13}\\  &  & -i{{\rm{\Omega }}}_{43}{e}^{i{\varphi }_{0}}{\rho }_{24}+i{{\rm{\Omega }}}_{32}^{\ast }({\rho }_{33}-{\rho }_{22}),\\ {\dot{\rho }}_{24} & = & -[i({{\rm{\Delta }}}_{32}+{{\rm{\Delta }}}_{43})+({\gamma }_{1}+{\gamma }_{3}\mathrm{)/2]}{\rho }_{24}+i{{\rm{\Omega }}}_{32}^{\ast }{\rho }_{34}\\  &  & +i{{\rm{\Omega }}}_{21}{\rho }_{14}-i{{\rm{\Omega }}}_{43}^{\ast }{e}^{-i{\varphi }_{0}}{\rho }_{23}-i{{\rm{\Omega }}}_{41}^{\ast }{\rho }_{21},\\ {\dot{\rho }}_{34} & = & -[i{{\rm{\Delta }}}_{43}+({\gamma }_{2}+{\gamma }_{3}\mathrm{)/2]}{\rho }_{34}+i{{\rm{\Omega }}}_{32}{\rho }_{24}\\  &  & +i{{\rm{\Omega }}}_{43}^{\ast }{e}^{-i{\varphi }_{0}}({\rho }_{44}-{\rho }_{33})-i{{\rm{\Omega }}}_{41}^{\ast }{\rho }_{31},\\ {\dot{\rho }}_{44} & = & -({\dot{\rho }}_{11}+{\dot{\rho }}_{22}+{\dot{\rho }}_{33}),\end{array}$$

where ${{\rm{\Delta }}}_{ij}={\omega }_{ij}-{\bar{\omega }}_{ij}$ defines frequency detuning between the applied laser field and |*i*〉 ↔ |*j*〉 transition and *ϕ*_0_ is the relative phase of the applied fields.

The generation of a fourth field with the frequency *ω*_41_ = *ω*_21_ + *ω*_32_ + *ω*_43_, known as FWM process, is a common phenomenon in which energy is completely conserved. Then, we study the response of the medium to the applied fields using the susceptibility of the |1〉 ↔ |4〉 transition which is proportional to the coherence term of the transition, *ρ*_41_. The assumption is that the positive imaginary part of the susceptibility is supposed to be the absorption response of the atomic system while its negative value indicates the gain. Note that the dispersion is determined by the real part of the *ρ*_41_ so that the positive (negative) slope of dispersion indicates the normal (anomalous) dispersion. In the special conditions, *γ*_1_ = *γ*_2_ = *γ*, *γ*_3_ = 0, Δ_32_ = Δ_43_ = 0, and Δ_41_ = Δ_21_ = *δ*, we obtain the following analytical expressions

3 $$\begin{array}{rcl}{\rho }_{21} & = & \frac{\mathrm{2[}{{\rm{\Omega }}}_{21}(i\gamma \delta -\mathrm{2|}{{\rm{\Omega }}}_{43}{|}^{2})+2{{\rm{\Omega }}}_{41}{{\rm{\Omega }}}_{32}^{\ast }{{\rm{\Omega }}}_{43}^{\ast }{e}^{-i{\varphi }_{0}}]}{{\gamma }^{2}\delta +2i\gamma |{{\rm{\Omega }}}_{43}{|}^{2}+4\delta (|{{\rm{\Omega }}}_{32}{|}^{2}+|{{\rm{\Omega }}}_{43}{|}^{2})},\\ {\rho }_{41} & = & \frac{4{{\rm{\Omega }}}_{21}{{\rm{\Omega }}}_{32}{{\rm{\Omega }}}_{43}{e}^{i{\varphi }_{0}}+\mathrm{(4}i\gamma \delta -{\gamma }^{2}-\mathrm{4|}{{\rm{\Omega }}}_{32}{|}^{2}){{\rm{\Omega }}}_{41}}{{\gamma }^{2}\delta +2i\gamma |{{\rm{\Omega }}}_{43}{|}^{2}+4\delta (|{{\rm{\Omega }}}_{32}{|}^{2}+|{{\rm{\Omega }}}_{43}{|}^{2})},\end{array}$$

for the coherence terms of the probe transitions, in the steady state. Now, we are going to find the amplitude of the GFF using the Maxwell equations. In the slowly varying envelope approximation and assumption of time-independent probe fields the Maxwell equations for two weak probe fields, propagating in *z* direction, read to

4 $$\begin{array}{rcl}\frac{\partial {{\rm{\Omega }}}_{21}(z)}{\partial z} & = & i\frac{\alpha \gamma }{2L}{\rho }_{21},\\ \frac{\partial {{\rm{\Omega }}}_{41}(z)}{\partial z} & = & i\frac{\alpha \gamma }{2L}{\rho }_{41},\end{array}$$

where *α* and *L* are the optical depth of the probe fields and atomic vapor cell length, respectively^22^. Considering the initial conditions for the probe fields amplitudes at the atomic medium entrance as Ω_41_(*z* = 0) = 0 and Ω_21_(*z* = 0) = Ω_21_(0), and *δ* = 0, simultaneous solving of equations (3) and (4) leads to an explicit term for the Rabi frequency of the GFF as

5 $${{\rm{\Omega }}}_{41}(z)=\frac{8{{\rm{\Omega }}}_{21}(0){{\rm{\Omega }}}_{32}{{\rm{\Omega }}}_{43}{e}^{i{\varphi }_{0}}}{A}\,{e}^{-\alpha z[{\gamma }^{2}+\mathrm{4(|}{{\rm{\Omega }}}_{32}{|}^{2}+|{{\rm{\Omega }}}_{43}{|}^{2})]/8L|{{\rm{\Omega }}}_{43}{|}^{2}}\,sinh(\alpha zA/8L|{{\rm{\Omega }}}_{43}{|}^{2}),$$

in which

$$A=\sqrt{\mathrm{16|}{{\rm{\Omega }}}_{32}{|}^{4}+{({\gamma }^{2}-\mathrm{4|}{{\rm{\Omega }}}_{43}{|}^{2})}^{2}+\mathrm{8|}{{\rm{\Omega }}}_{32}{|}^{2}({\gamma }^{2}+\mathrm{4|}{{\rm{\Omega }}}_{43}{|}^{2})},$$

and

$${{\rm{\Omega }}}_{32}={{\rm{\Omega }}}_{32}(r){e}^{il\phi }={{\rm{\Omega }}}_{{0}_{32}}\frac{1}{\sqrt{|l|!}}{(\frac{\sqrt{2}r}{{w}_{LG}})}^{|l|}\,{e}^{-{r}^{2}/{w}_{LG}^{2}}{e}^{il\phi },$$

at *z* = 0. According to equation (5), the phase factor of the strong coupling LG field transfers to the GFF, in the presence of two other planar fields. The planar microwave field has a key role in the OAM transfer so that there will not be the GFF in the absence of the microwave field. On the other hand, we would like to investigate the group velocity of the generated pulse inside the atomic vapor cell. In a dispersive medium, the different frequency components of the light pulse feel different refractive indices which affect the group velocity of pulse. The group velocity of a pulse is given by ${v}_{g}=|{\partial }_{\omega }\nabla {\rm{\Phi }}{|}^{-1}$, where Φ stands for the phase factor of the propagating pulse. In the proposed atomic model, the group velocity of the GFF at maximum intensity ring, ${r}_{max}={w}_{LG}\sqrt{|l|/2}$, is given by

6 $${v}_{g}(z)=\frac{c{(4{c}^{2}{z}^{2}+{w}_{LG}^{4}{\omega }^{2}{n}^{2})}^{3}}{(n+\omega \frac{\partial n}{\partial \omega })B},$$

with

$$\begin{array}{ccc}B & = & (16{c}^{4}{[-16{c}^{4}{r}_{max}{z}^{5}+{r}_{max}{w}_{LG}^{8}z{\omega }^{4}{n}^{4}]}^{2}\\  &  & +\,[-32{c}^{6}{z}^{4}({r}_{max}^{2}+(l+1){w}_{LG}^{2}-2{z}^{2})\\  &  & +\,48{c}^{4}{w}_{LG}^{4}{z}^{2}{\omega }^{2}{n}^{2}({r}_{max}^{2}+{z}^{2})+2{c}^{2}{w}_{LG}^{8}{\omega }^{4}{n}^{4}(-{r}_{max}^{2}\\  &  & {+(l+1){w}_{LG}^{2}+6{z}^{2})+{w}_{LG}^{12}{\omega }^{6}{n}^{6}]}^{2}{)}^{1/2},\end{array}$$

where *ω* is the GFF frequency. Here, the refractive index can be approximated in terms of the real part of the susceptibility as $n\approx 1+\chi ^{\prime} /2$. According to equations (3) and (6), the group velocity of a pulse can exceed the velocity of light in vacuum, leading to the superluminal light propagation. It is worth to note that, unlike the plane wavefront fields, the superluminal region for the LG pulses does not only determine by the anomalous dispersion and the OAM of the LG field affects the group velocity so that it may generate the superluminal light propagation even in the normal dispersion. On the other hand, the gain-assisted superluminal light propagation is the necessary condition for the OAM transfer to the GFF. The imaginary part of *ρ*_41_, at the entrance of the atomic medium, *z* = 0, and under the multi-photon resonance condition, *δ* = 0, is given by

7 $$Im({\rho }_{41})=\frac{-2{{\rm{\Omega }}}_{21}{{\rm{\Omega }}}_{32}(r){{\rm{\Omega }}}_{43}\,\cos (l\phi +{\varphi }_{0})}{\gamma |{{\rm{\Omega }}}_{43}{|}^{2}}\mathrm{.}$$

Then, the gain region in the absorption profile is determined by

8 $${{\rm{\Omega }}}_{32}(r)\cos (l\phi +{\varphi }_{0}) > 0.$$

## Results and Discussions

In this section, we present the results of our analytical calculations describing the OAM transfer from the strong coupling LG beam to a weak plane-wave via a weak planar microwave field. Moreover, we study the group velocity of the generated pulse, based on the equation (6), in the proposed atomic model. So, we focus on the coherence term *ρ*_41_, to investigate the spatially dependent absorption, intensity and phase profiles and group velocity of the microwave-induced GFF. All Rabi frequencies are scaled by *γ* = 2*π* × 6 *MHz*.

**Figure 2:**
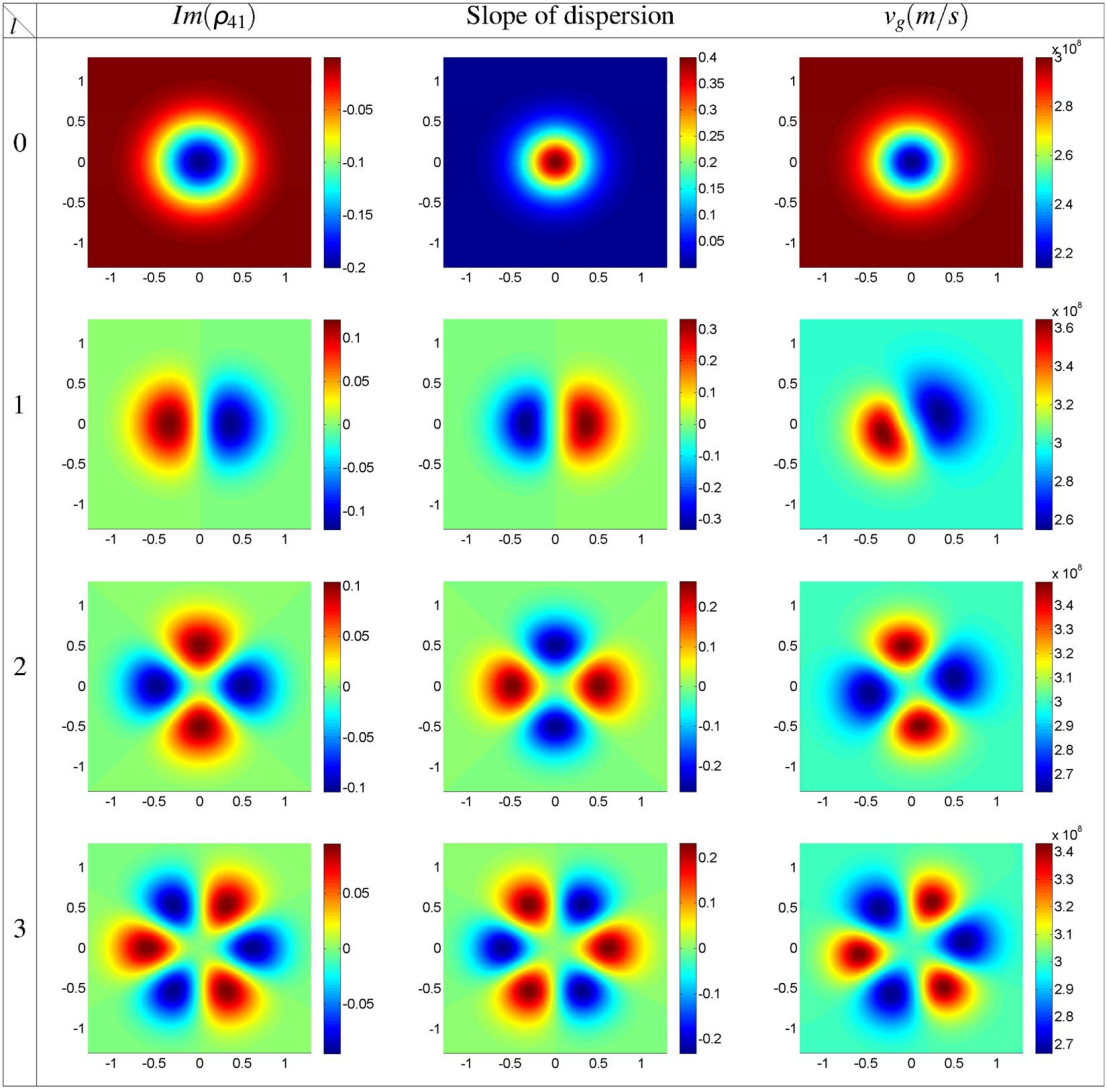
Imaginary part of *ρ*_41_, slope of dispersion, and GFF group velocity profiles as a function of *x* and *y* for strong coupling Gaussian field, *l* = 0, and different modes of the strong coupling LG field with *l* = 1, 2, 3 at the entrance of the atomic medium, *z* = 0. The horizontal, *x*, and vertical, *y*, axes are taken in *mm*. Used parameters are Δ_32_ = Δ_43_ = 0, Δ_41_ = Δ_21_ = *δ* = 0, *ω* = 788.7 *THz*, *ϕ*_0_ = 0, *w*_*LG*_ = 0.5 *mm*, Ω_21_(0) = 0.1*γ* and ${{\rm{\Omega }}}_{{0}_{32}}={{\rm{\Omega }}}_{43}=10\gamma$.

**Figure 3:**
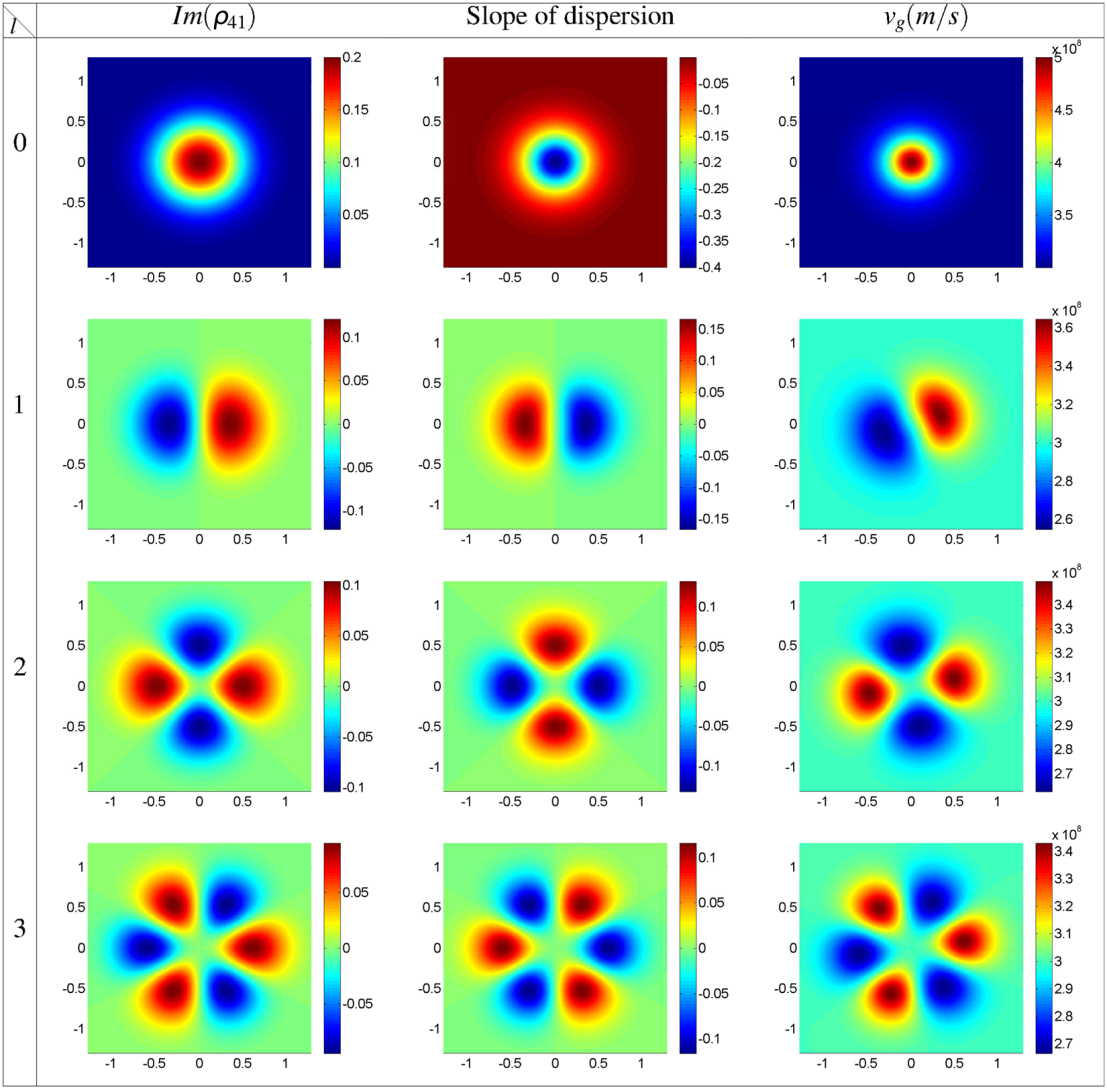
Imaginary part of *ρ*_41_, slope of dispersion, and GFF group velocity profiles as a function of *x* and *y* for strong coupling Gaussian field, *l* = 0, and different modes of the strong coupling LG field with *l* = 1, 2, 3 at the entrance of the atomic medium, *z* = 0. Other used parameters are same as in Fig. 2 except for *ϕ*_0_ = *π*.

Firstly, in Fig. 2, we plot the imaginary part of *ρ*_41_ (left column), slope of dispersion (middle column), and GFF group velocity (right column) profiles as a function of *x* and *y* for strong coupling Gaussian field, *l* = 0, and different modes of the strong coupling LG field with *l* = 1, 2, 3 at the entrance of the atomic medium, *z* = 0. The horizontal, *x*, and vertical, *y*, axes are taken in *mm*. Used parameters are Δ_32_ = Δ_43_ = 0, Δ_41_ = Δ_21_ = *δ* = 0, and *ω* = 788.7 *THz*. Other characteristics of the applied fields are chosen to be *w*_*LG*_ = 0.5 *mm*, Ω_21_(0) = 0.1*γ*, and ${{\rm{\Omega }}}_{{0}_{32}}={{\rm{\Omega }}}_{43}=10\gamma$. The spatially-dependent imaginary part of *ρ*_41_ profile shows a petal-like pattern with 2*l* number of petals with the gain regions which are compatible with equation (8). The left and middle columns of Fig. 2 are in good agreement with Kramers-Kronig relations. An investigation on the middle and right columns of Fig. 2, shows that the positive slope of dispersion always results in the subluminal group velocity for the strong coupling Gaussian field with a plane wavefront. But, for the strong coupling LG field with a helical wavefront, the positive (negative) slope of dispersion does not guarantee the subluminal (superluminal) light propagation. The right column of Fig. 2 shows that there are some regions of high absorption (gain) with superluminal (subluminal) group velocity. A small rotation in group velocity petal-like patterns, due to the OAM of the strong coupling LG field, prepares an unusual situation in which the positive (negative) slope of dispersion generates a superluminal (subluminal) GFF light propagation in the atomic system. This result persuaded us to investigate the group velocity behavior of the microwave-induced GFF and imaginary part of *ρ*_41_, further in its propagation direction inside the atomic system. In Fig. 3, we show the imaginary part of *ρ*_41_ (left column), slope of dispersion (middle column), and GFF group velocity (right column) profiles for *ϕ*_0_ = *π*. Other parameters are the same as in Fig. 2. It is shown that, by changing the relative phase of applied fields, the gain regions are changed into the absorption; so the slope of dispersion switches from positive to negative and vice versa. Then the subluminal and superluminal group velocity regions replace with together.

**Figure 4:**
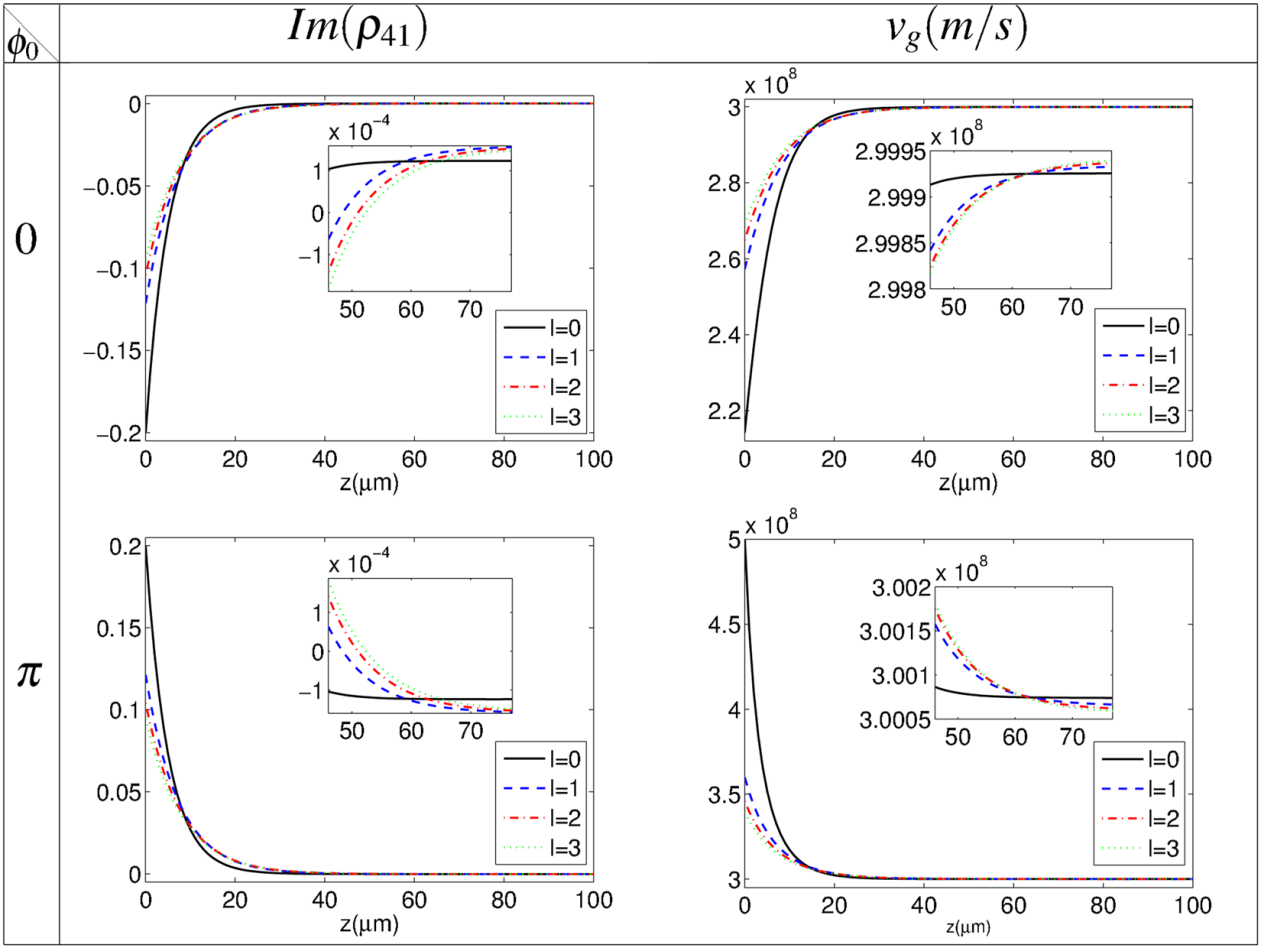
Imaginary part of *ρ*_41_ and GFF group velocity behaviors versus of *z* for strong coupling Gaussian field, *l* = 0, and different modes of the strong coupling LG field with *l* = 1, 2, 3 and two selected relative phases *ϕ*_0_ = 0, *π*, at *r*_*max*_. The other used parameters are the same as in Fig. 2.

Figure 4 shows the imaginary part of *ρ*_41_ (left column) and group velocity behavior of the microwave-induced GFF (right column) versus *z* for strong coupling Gaussian field, *l* = 0, and different modes of the strong coupling LG field, *l* = 1, 2, 3, in two relative phases, *ϕ*_0_ = 0, *π*, at *r*_*max*_. The other used parameters are the same as in Fig. 2. It is found that, by propagating the microwave-induced GFF in *z* direction, the negative imaginary part of *ρ*_41_ switches to the EIT with a small absorption which is accompanied by subluminal group velocity. Moreover, a switching between the absorption-assisted superluminal and the gain-assisted superluminal EIT window happens for *ϕ*_0_ = *π*. Note that the GFF attenuates during its propagation in the presence of the absorption and the OAM cannot effectively transfer from the strong coupling LG beam to the GFF. However, such disadvantage can be removed by changing the relative phase to *ϕ*_0_ = *π*. Then, the OAM transfer can be simply controlled by changing the relative phase of the applied fields in the closed-loop proposed atomic system.

**Figure 5:**
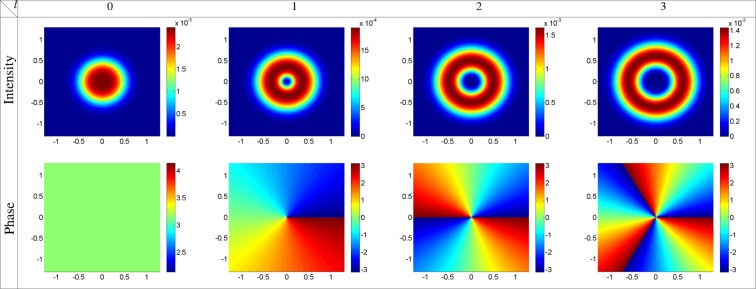
Intensity and phase profiles of the GFF at the end of the atomic vapor cell, *z* = *L* = 100 *μm*, as a function of *x* and *y* for the strong coupling Gaussian field, *l* = 0, and the first three modes of the strong coupling LG field, *l* = 1, 2, 3, *α* = 10, *δ* = 0, and *ϕ*_0_ = *π*, with the same parameters as used in Fig. 2.

**Figure 6:**
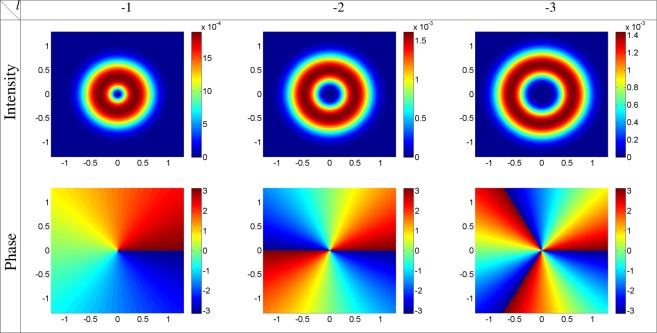
Intensity and phase profiles of the GFF at *z* = *L* = 100 *μm* versus *x* and *y* for different modes of the strong coupling LG field with the negative OAMs, *l* = −1, −2, −3, in which other parameters are same as in Fig. 5.

Let us now, study the optical properties of the GFF for *ϕ*_0_ = *π*. Using equation (5), the intensity and phase profiles of the GFF as a function of *x* and *y* are plotted at the end of the atomic vapor cell, *z* = *L* = 100 *μm*, for the strong coupling Gaussian field, *l* = 0, and the first three modes of the strong coupling LG field, *l* = 1, 2, 3, *α* = 10, *δ* = 0, and *ϕ*_0_ = *π* in Fig. 5, with the same parameters as used in Fig. 2. As expected from equation (5), the intensity and phase of the GFF follow the strong coupling field properties. The GFF is a Gaussian field with a constant phase when the strong coupling field is a Gaussian field. By choosing the strong coupling field as an LG field, the GFF becomes an LG field with the same OAM of the strong coupling LG field. The OAM transfer for the negative OAMs, *l* = −1, −2, −3, is illustrated in Fig. 6, in which other parameters are same as in Fig. 5. Overall, our model introduces a microwave-induced OAM transfer between the light beams via FWM process which is accompanied by the gain-assisted superluminal pulse propagation.

## Conclusion

In conclusion, we have theoretically investigated the microwave-induced OAM transfer and the group velocity of the GFF in a four-level ladder-type atomic system. It has been shown that the weak planar microwave field induces an OAM transfer from a strong coupling LG field to the GFF via FWM process. Moreover, we obtained an analytical expression to describe the GFF group velocity for different values of the OAMs. In addition, it has been found that the relative phase of the applied fields has a crucial role in controlling the OAM transfer as well as the group velocity of the GFF. We found a proper relative phase which switches the subluminal to the superluminal GFF propagation. The OAM transfer accompanied by the gain-assisted superluminal pulse propagation can be used in the development of optical communication, quantum information processing, and data transfer in high-dimensional Hilbert space.
